# Molecular Characterization and Pathogenicity Analysis of Novel Goose Parvovirus Isolated in Shandong Province Provide Insights into Viral Epidemic Tendency and Genetic Basis for Cross-Species Transmission and Pathogenicity Attenuation

**DOI:** 10.3390/ani15182696

**Published:** 2025-09-15

**Authors:** Yueyan Huang, Yuzhou Wang, Yaling Ding, Junkun Wang, Xiaojie Gao, Lin Zhang

**Affiliations:** 1 Institute of Animal Science and Veterinary Medicine, Shandong Academy of Agricultural Sciences, Jinan 250100, China; hyy10091999@163.com (Y.H.); wjk010113@163.com (J.W.); gxj091283@163.com (X.G.); 2 Key Laboratory of Livestock and Poultry Multi-Omics of MARA, Jinan 250100, China; 3 Nankai Animal Resources Center, State Key Laboratory of Medicinal Chemical Biology, Nankai University, Tianjin 300071, China; wangyuzhou@naikai.edu.cn; 4 Qilu Animal Health Products Co., Ltd., Jinan 250100, China; dingyaling1114@163.com

**Keywords:** novel goose parvovirus, genetic evolution, diversity, cross-species transmission, pathogenicity

## Abstract

The novel goose parvovirus (NGPV), an agent of short beak and dwarfism syndrome (SBDS), has been circulating in China since 2015; however, the absence of systematic genetic evolution and diversity analysis has limited our understanding of the viral epidemic tendency and genetic basis for cross-species transmission and pathogenicity attenuation. In this study, five NGPVs were isolated in 2022–2023, and their genomes were sequenced. A comprehensive analysis of genetic evolution and diversity was conducted using sequences of representative waterfowl parvoviruses isolated at different periods. Subsequently, pathogenicity and sero-response studies in ducks were also conducted. The results indicated that circulating NGPV in China could be divided into two genotypes and two subtypes and three epidemic phases from evolutionary and temporal dimensions, respectively. Currently, NGPV Ia is the dominant genotype across China. Phylogenetic analysis demonstrated that VP1 was the leading factor in viral evolution. The genetic diversity analysis showed regular mutation sites in both proteins. According to their location and resultant structural changes, combining the results of pathogenicity and humoral immune response, several substitutes are possibly latent residues for the virus to achieve cross-host infection, escape from antibody recognition, and undergo pathogenicity alteration. The findings of this study provide novel insights into the viral epidemic tendency and genetic basis for cross-species transmission and pathogenicity attenuation and will help to facilitate the development of vaccines and antiviral therapy.

## Simple Summary

Since 2015, the outbreak and spread of novel goose parvovirus (NGPV) has caused huge economic losses to the duck industry; however, in the absence of systematic genetic evolution and diversity analysis, commercial vaccines have never been developed. Moreover, the molecular basis of some phenomena was not clear, including NGPV spillover to duck from goose, goose parvovirus (GPV) antibodies not effectively neutralizing NGPV, and the pathogenicity of NGPV being significantly attenuated compared with earlier circulating strains. Therefore, we conducted a systematic and detailed genetic evolution and diversity analysis with known NGPV genomes that ran through different epidemic stages. Combined with protein reconstruction, animal regression, and antibody detection, the epidemic tendency and potential factors for cross-species transmission and pathogenicity attenuation were identified. These results provided a solid foundation for the development of effective vaccines and therapeutic drugs, as well as a basis for clarifying the source of NGPV.

## 1. Introduction

Waterfowl parvoviruses belong to Anseriform dependoparvovirus I of the genus Dependovirus and family Parvoviridae. These viruses contain linear and single-stranded DNA genomes approximately 5 kb in size and consist of two untranslated regions (UTRs) with hairpin structures and two open reading frames (ORFs) (NS1 and VP1) [1]. Waterfowl parvoviruses are classified into goose parvovirus (GPV) and Muscovy duck parvovirus (MDPV) [2]. GPV primarily infects geese and Muscovy ducks, causing Derzsy’s disease characterized by watery diarrhea and high mortality, whereas MDPV only affects young Muscovy ducklings and causes ‘three-week’ disease, characterized by enteritis, myocarditis, hepatitis, and high mortality [3].

Vaccines against GPV and MDPV have been extensively utilized in cultivating waterfowl, leading to a noticeable reduction in disease incidence [4]. However, owing to constant mutation and recombination under multiple selection pressures, such as evolution and vaccines, variant viruses undergo changes in pathogenicity and have the potential to spill over from the original host, leading to new epidemics. Early in the 1970s–1980s, distinct GPVs infected mule ducks in Europe and all breeds of ducks in Taiwan. Infected ducks manifested bill atrophy syndrome with atrophy of the upper bills, protruding tongue, and stunting [5,6]. In 2009, mule ducks in Europe experienced another outbreak of short beak and dwarfism syndrome (SBDS) caused by a distinct lineage of GPV, with variations in susceptible duck species and pathogenicity [6]. In mainland China, a variant of MDPV caused SBDS in mule and Taiwan white ducks without tongue protrusion in 2008, indicating that MDPV can infect other breeds of ducks [3]. However, these evidences of cross-host transmission of waterfowl parvoviruses remained partially ignored until 2015, when an outbreak of SBDS, characterized by short beaks, protruding tongues, and stunts, occurred in several duck farms in China. Following the pandemic, viral isolation, identification, and experimental infections were carried out in several studies, and the pathogen, a novel goose parvovirus (NGPV), was confirmed [7,8,9]. Owing to the lack of corresponding prevention technologies and products, significant economic losses have seriously hindered the healthy development of the waterfowl industry. Yet, to date, effective vaccines or treatments to prevent this disease remain lacking.

Genetic mutations are the basis of changes in protein structure and function. Gene analysis can provide deep insights into the mechanisms underlying viral evolution and spread, as well as the origin of disease outbreaks [10,11]. Currently, there are some available data on the epidemiology, isolation, genetic diversity, and recombination of NGPV [12,13,14,15]. However, the lack of systematic genetic evolution and diversity analyses has created a bottleneck for elucidating the viral epidemic tendency and genetic basis for cross-species transmission and pathogenicity attenuation.

In this study, we isolated five NGPVs in Shandong province from 2022 to 2023 from infected ducks with growth restriction, along with feather and leg loss fractures. Following genome sequencing, genetic evolution and diversity analyses were conducted with known sequences of waterfowl parvoviruses to illustrate the NGPV epidemic tendency and genetic evolution. The findings will provide fundamental data to facilitate the development of effective vaccines and therapeutic drugs.

## 2. Materials and Methods

### 2.1. Sample Collection and Treatment

During 2022–2023, infected ducks in several large scale duck farms in Shandong province exhibited decreased appetite, slow growth, paralysis, and depilation. The morbidity and mortality rate were 5–8% and about 1%, respectively, and major postmortem features include swollen liver and kidney, splenic necrosis, and turbid air floats. Diseased organs, including the liver, kidney, spleen, intestine, and heart, were collected from infected ducks on five farms distributed across different districts and counties and transported to the laboratory for further treatment. Tissue samples were homogenized in phosphate-buffered saline (PBS) (pH 7.2) to obtain a 10% suspension (*w*/*v*) and centrifuged at 12,000 rpm (4 °C) for 10 min. The supernatant was collected and stored at −80 °C until further use.

### 2.2. Virus Detection

Viral DNA/RNA was extracted using the Viral DNA/RNA Kit (Bioer Technology Hangzhou, China) and used as a template for polymerase chain reaction/real-time polymerase chain reaction (PCR/RT-PCR) detection, as previously described [11]. The viruses detected included duck tembusu virus (DTMUV), duck hepatitis A virus (DHAV), waterfowl parvovirus (WPV), aviadenovirus (AAV), duck plague virus (DPV), avian influenza virus (AIV), Newcastle virus (NDV), duck astrovirus (DAstV) and duck reovirus (DRV). PCR products were separated using agarose gel electrophoresis, and positive ones were sequenced by Sangon Biotech (Shanghai, China).

### 2.3. NPGV Isolation and Purification

NGPV-positive supernatant was filtered through a 0.22 µM filter and incubated with penicillin (1000 IU/mL) and streptomycin (1000 µg/mL). Viral inoculation (200 μL) was used to inoculate 9-day-old specific-pathogen-free (SPF) duck eggs (Jingding duck, purchased from Shandong Health-tech Laboratory Animal Breeding Co., Ltd. (Jinan, China)) via the allantoic avity route, which continuously propagated for 3–4 generations. NGPV and other viruses mentioned above were detected by PCR/RT-PCR followed allantoic fluid of each passage collected. After the embryos’ death was fixed, the allantoic fluid was pooled to purify the virus using a limited dilution method, as previously described [16]. The purified virus was serially diluted 10-fold, and 200 µL of each dilution was subjected to 9-day-old SPF duck eggs (*n* = 5). Subsequently, the eggs were candled for 7 days at 37 °C and the numbers of dead eggs recorded at each dilution. Finally, the ELD50 assay was carried out using the Reed-Muench method.

### 2.4. Genome Amplification and Sequencing

To amplify the NGPV genome, specific primers (Table 1) were designed according to known NGPV sequences retrieved from the NCBI database, and the segments were amplified as previously described [11]. PCR products were extracted and cloned into pMD18-T (TaKaRa Bio, Dalian, China), and then transformed into competent *Escherichia coli* DH5α (Transgen Biotech, Beijing, China). Positive clones identified using PCR were sequenced by Sangon Biotech (Shanghai, China). Finally, all the sequenced segments were joined to the genome.

### 2.5. Genetic Evolution and Mutation Analysis

First, a phylogenetic analysis of the genomes, ORFs, and deduced amino acid sequences of waterfowl parvoviruses (GPV, NGPV, and MDPV), including the five NGPV isolated in this study, was conducted. Sequence alignment was performed using MAFFT, and a phylogenetic tree was constructed using MEGA6.0. The maximum likelihood tree was established using the GTR model based on the gamma distributed. Bootstrap values were estimated for 1000 replicates. Second, an adaptive analysis of the NGPV ORF sequences was performed. Based on the ratios of non-synonymous to synonymous (*d*_N_/*d*_S_), the mixed-effects model of evolution (MEME) [17], single-likelihood ancestor counting (SLAC) [18], and fast unconstrained Bayesian approximation (FUBAR) [19] were employed to predict positive selection sites during evolution. The residues were considered positive selection sites only if supported by at least two algorithms. Finally, nucleotide and amino acid sequence identities between the five new NGPV isolates and representative sequences belonging to different genotypes (subtypes) were determined using DANMAN 5.2.2 (Lynnon). Variant amino acids were selected and marked using JalView [20], and the key substitutions in VP1 were located in the 3D structure. Protein structure homology modeling and highlights were performed using SWISS-MODEL (https://swissmodel.expasy.org/, accessed on 7 October 2024) and PyMOL (version 3.1.6.1), respectively.

### 2.6. NGPV Pathogenicity and Serology in Ducklings

According to the result of ELD_50_ determination, isolates with higher titers (≥10^5^) (D271 and D278) were selected to assess pathogenicity in ducklings. A total of 75 healthy SPF ducks (3-day-old) were randomly divided into three groups, each containing 25 ducks. Groups infected with D271 and D278 (5 × 10^5^ ELD_50_) via the intramuscular route were named 271 and 278, respectively, and the control group was injected with equal amounts of PBS. Each group was individually reared in a room for routine feeding and management. The experiment lasted 70 days, and clinical manifestations and deaths were recorded daily. At 1, 4, 7, 14, 21, 28, 35, and 70 days post-infection (dpi), peripheral blood was collected from ducks in each group and Ab titers were determined using the agar gel precipitation (AGP) test, in which GPV and NGPV isolated and conserved in our laboratory were used as antigens. Abs titers were expressed as the log2 of the reciprocal of the highest serum dilution, producing a precipitation line. At each time point, three birds from each group were sacrificed. The heart, liver, spleen, lungs, kidneys, stomach, pancreas, duodenum, bursa, and skeletal muscle were collected, and the lesions were observed. NGPV loads in different organs or tissues were measured using quantitative RT-PCR (RT-qPCR) as previously described [21]. Each sample was analyzed in triplicate.

### 2.7. Statistical Analysis

The data of viral copies in different tissues and Ab titers at different times were analyzed using two-way ANOVA and one-way ANOVA, respectively, according to Dunn’s multiple comparison procedure in GraphPad Prism 8.0. The mean values were considered statistically significant at *p* < 0.05. The data are expressed as the mean and pooled SEM.

## 3. Results

### 3.1. Isolation and Sequencing of NGPV

Of the five samples collected from different duck farms, only NGPV was detected. Supernatants containing NGPV were incubated in 9-day-old SPF duck eggs; after 3–4 passages, the embryos were lethal at relatively fixed time points (Table 2). Further limited dilutions were performed to obtain the corresponding NGPV isolates, referred to as D187, D271, D272, D274, and D278. D271 was the most virulent in duck embryos, leading to death at 48 h, whereas D278 had the weakest virulence, with a death time of 144 h; the remaining three strains showed variations between the two isolates. Subsequently, ELD_50_ of all isolates was determined. Titers of D271 and D278 were higher, reaching 10^−6.32^/0.2 mL and 10^−6.16^/0.2 mL, respectively, while the others were relatively lower (Table 2).

The genomes of the five NGPV samples isolated in this study were joined from the segments amplified using specific primers and submitted to GenBank (Table 2). The results showed that PQ807633-PQ807637 had components similar to those of known NGPV. The genomes were 5052 base pairs (bp) long (5022 bp in D274) and contained two ORFs and two UTRs (5′UTR and 3′UTR) flanking the ends of the genomes. The left ORF (513–2396 bp) encoded the NS protein, which was 627 amino acids (aa) and was spliced into NS1 (627 aa) and NS2 (451 aa) proteins using a common stop codon. The right ORF gene (2415–4613 bp) encodes the VP protein, which is 732 aa and can be cut into VP1 (732 aa), VP2 (587 aa), and VP3 (534 aa). Similarly to NS1 and NS2, VP1-VP3 also used the same stop codon. There was a 19 nt long intergenic region between the NS stop codon and the VP start codon. In the 5′UTR and 3′UTR, the inverted terminal repeats (ITRs) were 382 and 376 bp, respectively (D274 had two deletions in this region at 4816–4830 bp and 4919–4933 bp, respectively, 30 bp in length). Similarly to other waterfowl parvovirus genomes, several transcription factor binding sites conserved were present in 5′UTR and 3′UTR, such as Y-box, E-box, major late transcription factor (MLTF), Rep protein binding site (RBS), activating transcription factor/cAMP response element binding protein (ATF/CREB) [22]. Not only in UTR, but also in NS1 and VP1, multiple functional domains were conserved: DNA binding motif (142–168 aa) [7], helicase superfamily 3 (SF3) domain (222–492 aa) [23], ATP binding region (292–386 aa) [24], NTP binding motif (336–343 aa) [9]. Transactivation domain (556–627 aa) [25] associated with viral replication was in NS1 protein, while phospholipase A2 (PLA2) (53–111 aa) and nuclear localization signal (NLS) (160–171 aa) [26] related to proliferation and localization were in VP1 protein.

### 3.2. Phylogenetic Analysis of NGPV Genes

**Figure 1:**
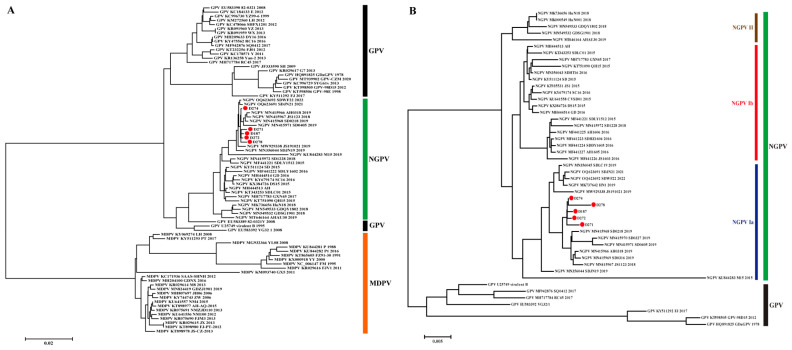
Phylogenetic analysis of NGPV based on genomic sequences. (**A**) Phylogenetic tree constructed using GPV, NGPV and MDPV genomes. (**B**) Phylogenetic tree constructed with GPV and NGPV genomes. GPV, MDPV and three genotypes/subtypes of NGPV were indicated. The viruses identified in this study were indicated by red circles.

A total of 78 complete genomes of representative waterfowl parvoviruses (GPV, MDPV, and NGPV) were used for phylogenetic analysis. The results showed that five strains isolated in the present study clustered with NGPV and formed the GPV branch with GPV. This branch was distant from the MDPV one, indicating that NGPV evolved from GPV and had a distant evolutionary relationship with MDPV (Figure 1A). A total of 48 GPV and NGPV genome sequences in the GPV branch were further analyzed, showing that the two viruses clustered separately, forming different branches. Focusing on the NGPV branch, viruses evolved into two clusters, genotypes I and II: genotype I could be further divided into two subtypes Ia and Ib (Figure 1B). The five strains isolated in this study and most NGPVs reported after 2017 belonged to NGPV Ia, suggesting that NGPV Ia is currently the dominant genotype in China.

**Figure 2:**
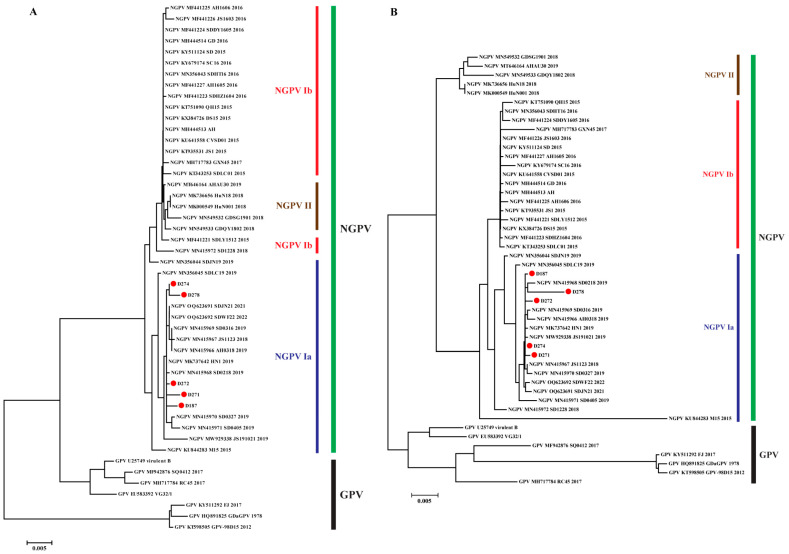
Phylogenetic analysis of NS1 (**A**) and VP1 (**B**) based on nucleotide sequences. GPV and the three genotypes/subtypes of NGPV are indicated. Viruses identified in this study are indicated by red circles.

To confirm the dynamics of NGPV formation of different genotypes and subtypes, nucleotide sequences of NS1 and VP1 were used to construct phylogenetic trees. As shown in Figure 2, the structure of the phylogenetic tree based on the VP1 nucleotide is similar to that generated using genomic sequences (except for MN415972), revealing that VP1 is the primary factor in viral evolution. Subsequently, 41 NGPV sequences were selected for adaptive evolutionary analysis, which determined the genetic factors involved in forming the different genotypes (subtypes). NS1 and VP1 mainly underwent negative selection, with *d*_N_/*d*_S_ being 0.38 and 0.40, respectively, although eight positive selected sites were identified. Among the selected sites, five were distributed in VP1, and the remaining were in NS1, further confirming the dominant role of VP1 in genetic evolution. Under selection pressure, the properties, particularly the polarity of these sites, underwent certain changes (four out of five non-polar sites transformed into polar sites) (Table 3).

**Figure 3:**
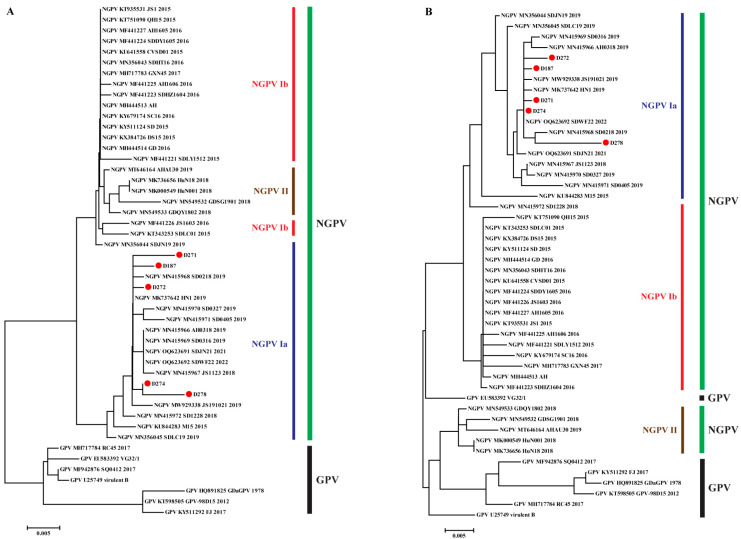
Phylogenetic analysis of NS1 (**A**) and VP1 (**B**) based on amino acid sequences. GPV and the three genotypes/subtypes of NGPV are indicated. Viruses identified in this study are indicated by red circles.

Non-synonymous mutations and positive selection sites lead to alternations in amino acids. Thus, the amino acid sequences of NS1 and VP1 were used to construct phylogenetic trees. As illustrated in Figure 3, the tree of NS1 was consistent with that constructed using nucleotide sequences (except for MN415972). NS1 proteins of GPV and NGPV were distributed in different branches, and in the NGPV branch, NGPV Ib and II clustered together, whereas NGPV Ia was relatively distant (Figure 3A). In contrast, there were significant changes in the amino acid phylogenetic tree of VP1 compared with that constructed based on nucleotides: NGPV II formed an evolutionary branch with GPV but was separated from NGPV I (Figure 3B). The results confirmed the mutation and evolution of VP1 drove NGPV differentiation into different genotypes and suggested that the two encoded proteins experienced different selection pressures and exhibited evolutionary patterns during evolutionary history.

### 3.3. Mutation Analysis of NGPV Proteins

To confirm whether the structural differences between the two phylogenetic trees (based on nucleotide and amino acid sequences) of specific genes depended on amino acid substitutions and adaptive selection residues, we conducted homology and mutation analyses of viral sequences. First, the nucleotide and amino acid sequence homology of the five newly isolated strains were compared with those of various NGPV genotypes/subtypes and representatives of GPV. The results indicated that the five new isolates had high homology at the nucleotide and amino acid levels with the others (Table 4). Specifically, at the nucleotide level, GPV had the lowest homology with the new isolates, followed by NGPV II, whereas, at the amino acid level, the lowest homology appeared for the NS1 protein of GPV and the VP1 protein of NGPV II, respectively.

**Figure 4:**
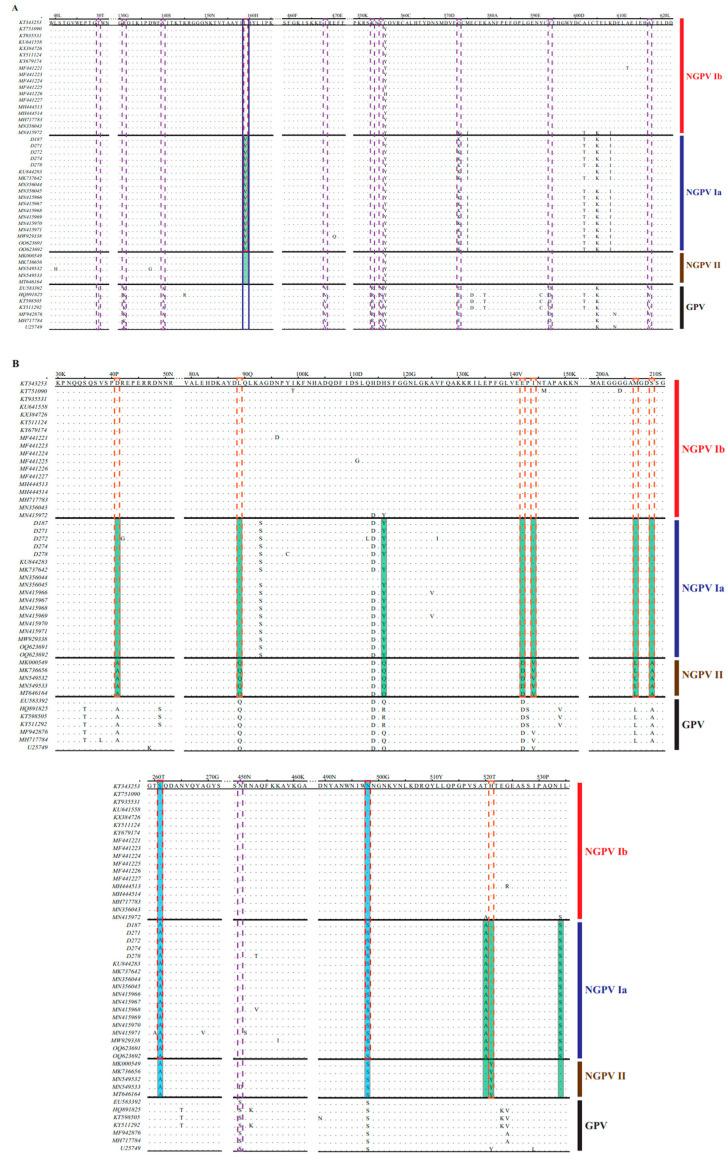
Mutational analysis of NGPV NS1 (**A**) and VP1 (**B**). Regular substitutions between GPV and NGPV are indicated by purple dashed boxes. Regular mutations between NGPV I and II are indicated by orange dashed boxes. Three regular substitutions between NGPV Ia and Ib are indicated by red dashed boxes. Characteristic substitutions considered as genetic markers in NGPV Ia are indicated by the blue box. Regular mutations in NGPV Ia and II are indicated in green. The two regular substitutions between NGPV isolated during 2015–2017 and strains after 2017 are indicated in blue. GPV and the three genotypes/sub-genotypes of NGPV are indicated.

Second, we screened 48 amino acid sequences of GPV and NGPV, including all genotypes, to identify mutations. As shown in Table 5 and Figure 4, several characteristic substitutions could be divided into four levels: (1) regular substitutions between GPV and NGPV, completely distinguished between each other, distributed in VP1 (1 residue) and mainly C terminal of NS1 (9 residues); (2) regular mutations between NGPV I and II (7 amino acids), distributed in VP1 protein, were completely conserved within respective genotypes; (3) three regular substitutions between NGPV Ia and Ib, located at positions 261 and 498 of VP1 and 159 of NS1, which were S, N and L in NGPV Ib, but mutated to A, S and V in NGPV Ia; (4) in NGPV Ia, the characteristic substitution at position 159 of NS1 was V, whereas in NGPV Ib/II and GPV, it was replaced by L. This alteration could serve as a genetic marker for NGPV Ia.

**Figure 5:**
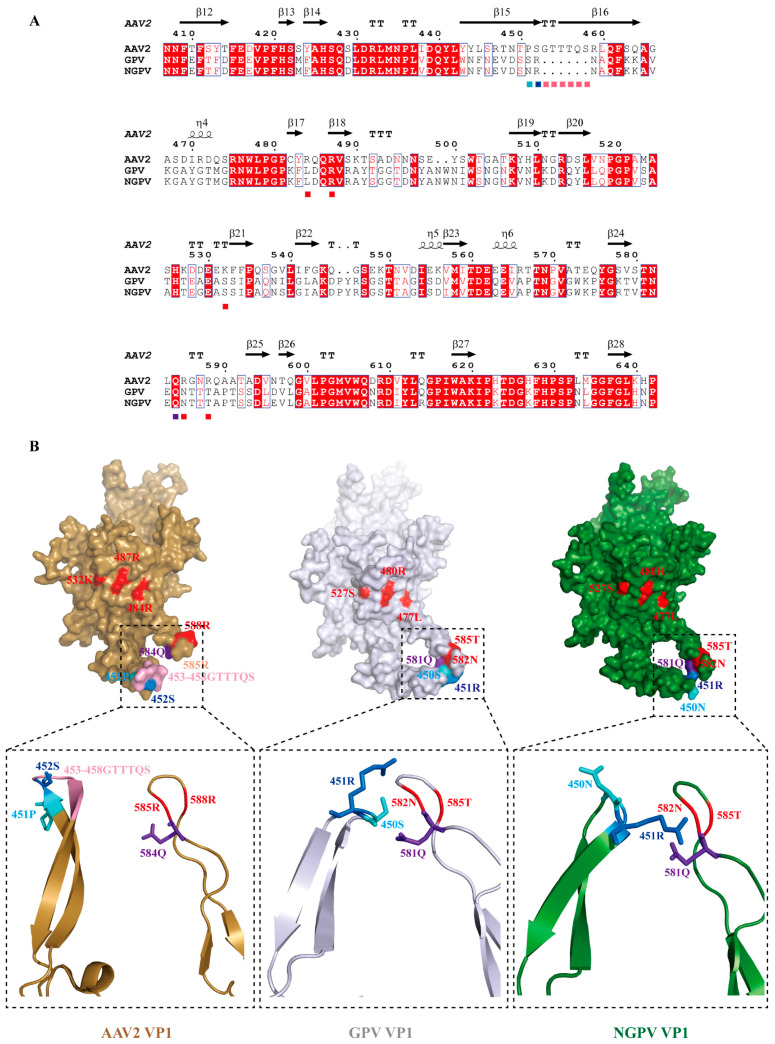
Comparative analysis of amino acids and structures of AAV2, GPV, and NGPV VP1 proteins. (**A**) Sequence alignment of VP1 of AAV2, GPV, and NGPV strains. The sequences were then truncated. Distribution of secondary structures is shown. The residues interacting with the receptor are indicated by red squares. Positions 451, 452, and 584 are indicated by the cyan, blue, and purple squares, respectively. Residues 453–458 are indicated by light pink squares. (**B**) Alignment display of the VP1 structures for AAV2, GPV, and NGPV. VP1 is shown as a surface representation, with different conformations mainly concentrated at the bottom of VP1. An enlarged view shows residues 451–458 and 584–588 (position in AAV2 VP1) displayed in an animated representation, with residues 451, 452, and 584 depicted as sticks. The structures of VP1 in AAV2, GPV, and NGPV are colored sand, gray, and forest, respectively. The residues (484, 487, 532, 585, and 588; numbering in AAV2 VP1) recognized by the receptor are indicated in red. Residues 451, 452, and 584 are indicated in cyan, blue, and purple, respectively. Residues 453–458 are indicated in light pink.

Finally, a VP1 homologous model of GPV and NGPV was constructed using AAV2 VP1 (PDB: 6U3Q) as a template, and the only regular mutation site was located. The results indicated that 450S/451R (corresponding to 451P/452S in AAV2 VP1) merged with 528N/585T (receptor-binding sites 585R/588R in AAV2 VP1) in the GPV VP1 protein, owing to several residue defects (Figure 5A). The 450S of GPV VP1 was further mutated to 450N in NGPV VP1, resulting in it protruding outward from the surface of the protein and making 451R closer to 581Q (Figure 5B). This conformational change may alter interactions between VP1 and its receptors.

### 3.4. Pathogenicity of NGPV Isolates to Ducklings

**Figure 6:**
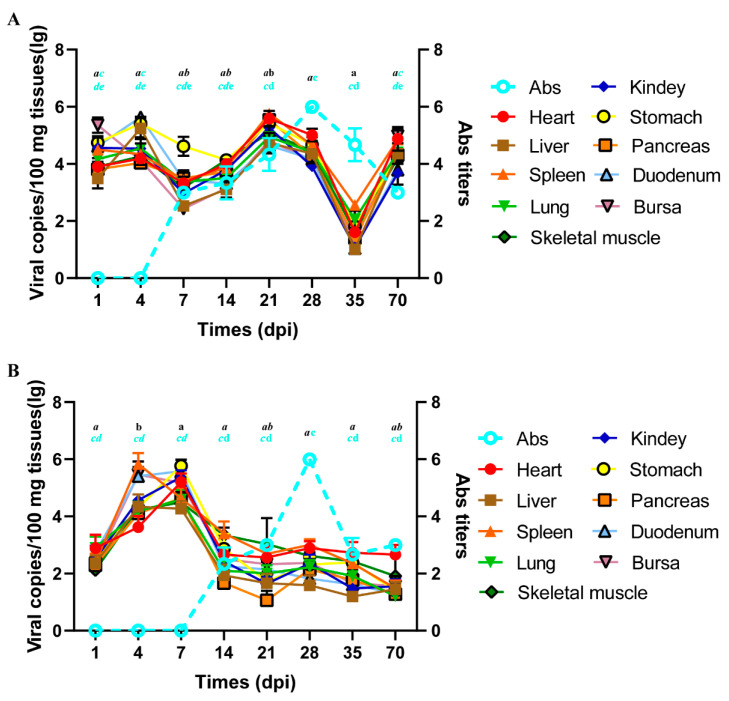
Serological and virological profiles of ducks experimentally infected with strains D271 (**A**) and D278 (**B**). Viral loads are indicated with lines that changed significantly in all detected items at the same point and were considered to be significantly different (*p* < 0.05). Comparative objects are indicated by the same letters in black, with different letters representing significant differences (*p* < 0.05). Antibody titers are indicated by dashed lines, and comparative objects are indicated by the same letters in cyan, with different fonts representing significant differences (*p* < 0.05).

Ducklings were challenged with D271 and D278. Most ducks were normal, and no deaths were observed following the viral attack. At 21 dpi, two ducks in group 271 manifested growth retardation and slight bleeding points in the intestine. However, until 56 dpi, two birds in group 278 had fractured feathers and protruding tongues, and slight bleeding was observed in the spleen and intestine but not in other organs. The viral loads in different organs and tissues at each time point were determined (Figure 6). The virus was distributed in all detected items, and the loads presented a ‘W’ shape during the experimental period in group 271, with lower viral content at 7–14 dpi and 35 dpi, but higher at the remaining time points. Specifically, viral loads peaked at 21 dpi, which was significantly different at 7, 14, and 35 dpi (*p* < 0.05) and then dropped to the lowest at 35 dpi (*p* < 0.05). In group 278, although all detected items were positive, the tendency with time differed from that in group 271. The viral loads were higher at 4 dpi (except for the heart) and 7 dpi (*p* < 0.05) but lower at the other time points (*p* > 0.05). No viruses were detected in the control group. Notably, at the same point, there was no significant regularities in the viral content among various organs and tissues, as well as differences in viral loads among most organs and tissues (*p* > 0.05). Thus, viral load in all detected items at the same point was regarded as a whole, and when it displayed a significant change, the significant difference could be determined.

Subsequently, we determined the titers of antibodies (Abs) against NGPV in peripheral blood at each time point. The Ab was detected at 7 dpi and gradually peaked at 28 dpi (*p* < 0.05), followed by a decrease until 70 dpi, equivalent to the 7–14 dpi level (*p* > 0.05) in group 271. By comparison, the Ab detected in group 278 was later (14 dpi), and titers peaked at 28 dpi (*p* < 0.05), which gradually decreased and maintained at 2^2^–2^3^. In the AGP test, precipitation lines that reacted with serum Abs against D271 and D278 were observed only when NGPV was the antigen. These results demonstrate no immunological cross-reactivity between GPV and circulating NGPV.

Compared with the pathogenicity of NGPV strains isolated during the early period, the pathogenicity of D271 and D278 in ducklings significantly decreased. To confirm the genetic basis, we analyzed the regular amino acid substitutions between different evolutionary periods. As shown in Table 6 and Figure 4, two regular mutations at positions 261 and 498 located in VP1 were present between the strains isolated during 2015–2017 (belonging to NGPV Ib, except KU844283) and those prevalent after 2017 (NGPV II and Ia, except MN415972). These may be the leading factors in attenuating the pathogenicity. Further analysis showed 11 other regular substitutions in VP1 (10 sites) and NS1 (1 site) between the NGPV II and Ia amino acid sequences, although both genotypes (subtypes) circulated after 2017. These mutations can be used as genetic markers to distinguish between the two genotypes (subtypes).

## 4. Discussion

SBDS, caused by NGPV infection, is a principal disease seriously hindering the development of the ducking industry. However, effective preventive measures and therapies have been lacking over the past 10 years. Vaccines are powerful tools for preventing viral infections. An efficient vaccine should possess high coverage to prevent the most prevalent strains [27] and have the long-term ability to guard against present and mutant strains for a considerable period [28]. To achieve these objectives, an in-depth understanding of viral epidemics and genetic variation patterns is essential, which was required in the analysis of virus epidemiology and sequences. Therefore, we sequenced the genomes of five NGPVs isolated during 202–2023 and conducted systemic genetic diversity and evolutionary analyses with sequences of GPV, MDPV, and NGPV isolated from different periods.

GPV, MDPV, and AAV2 share a common ancestral origin [22], whereas NGPV belongs to a distinct lineage of GPV [7]. Therefore, NGPV should have high genetic homology and present structural composition and functional motifs similar to those of the aforementioned viruses. This hypothesis was confirmed by sequence alignment and analysis of five new isolates. Consistent with previous studies [4], the genome structure of NGPV isolated in the present study was relatively conserved, with no wide range deletions or insertions, while, except in D274, there was 30 bp missing in the 3′UTR that resulted in the defect of two MLTFs. According to the results of RNA structure prediction, defective segments did not affect the formation of U-shaped structures or the replication function of the virus [4]; however, the shortened distance between functional regions might influence viral transcription efficiency (especially in the late stages) and virulence. In addition, some studies have indicated that the ITR length positively correlates with virulence [29]. However, in terms of toxicity to duck embryos, the shortening of the ITR in strain D274 did not weaken its virulence.

Genetic evolution analysis indicated that NGPV can be divided into two genotypes, with NGPV I further evolving into two subtypes. Combined with the time dimension, the prevalence of NGPV in China could be divided into three periods: early period (2015–2017), in which NGPV Ib was the dominant genotype; middle period (2018–2019), when NGPV Ia and II co-circulated; recent period (after 2019), when NGPV Ia was the dominant genotype (containing the five newly isolated NGPVs). Therefore, NGPV Ia became the dominant genotype prevalent in China and shifted the priority to prevention and vaccine development. A phylogenetic tree and adaptive evolution of different NGPV ORFs were constructed to confirm the key drivers of viral mutation and evolution. The phylogenetic tree constructed with the NS1 nucleotide was similar to that constructed using amino acids, directly reflecting the relatively conservative characteristics of NS1. The result was also consistent with the lower *d*_N_/*d*_S_ values and fewer positive selection sites. Moreover, according to the results of NGPV Ib and II co-evolving and NGPV Ia evolving separately, NS1 had already differentiated into two major branches (Ib/II and Ia) during the early period of NGPV prevalence and was mainly NGPV Ib/II. However, with the sustained effect of selection pressure, NGPV Ia gradually became dominant (Figure 2A and Figure 3A). Instead, the structure of the phylogenetic tree based on the VP1 nucleotide was similar to the tree of genome sequences, indicating that VP1 is the leading factor in viral evolution and the higher *d*_N_/*d*_S_ value and more positive selection sites (compared with NS1) indicated the basis of the differences between amino acid and nucleotide phylogenetic trees and confirmed its driving role in genetic evolution. Notably, the homology of VP1 between GPV and NGPV was high; however, an individual branch was formed by NGPV II and GPV in the amino acid phylogenetic tree. We could not determine whether the result represented the characteristics of reverse mutation in NGPV, as over the past few decades, the epidemic caused by GPV was intermittent outbreaks and not enough genes were obtained during the early period [5,6]. However, the data suggested that there were two different evolutionary directions in the evolutionary history of the NGPV VP protein.

The genotype determination of circulating viruses is critical for vaccine development with high coverage, whereas the regularity determination of viral genetic variation is the key to vaccine availability. Therefore, we analyzed the regular amino acid substitutions in NGPV. Recombination analysis was conducted prior to mutation analysis; no recombination events occurred in the five new isolates, although several characteristic mutations were found. The mechanism by which GPV spills over into ducks remains unclear. Binding to cell receptors is a prerequisite for acquiring the ability to infect new hosts [30]. Thus, sequence and subsequent structural alterations of the receptor-binding protein (RBP) facilitate the invasion of NGPV into duck-derived cells. VP1, containing the major structural protein VP3 with the greatest number and combined with the receptor, is the primary protein recognized by the cell receptor in parvoviruses [26]. However, due to the absence of the 3D structure of GPV and NGPV VP1, AAV2 VP1 is often used as a modeling structure to determine the changes in structure caused by residue mutations. In comparison, the structural differences in VP1 between AAV2 and waterfowl parvoviruses were mainly concentrated close to 585R and 588R, forming canyons where the virus–cell receptor interaction occurred with 484R, 487R, and 532K in another adjacent VP1 protein [31]. Compared with AAV VP1, the deletion of 453G-458S (numbering in AAV2 VP1), made 450S and 451R (corresponding to 451P and 452S in AAV2 VP1) in GPV VP1 and 451R located in NGPV VP1 fuse with 581Q and 582N (numbering relative to 584Q and 585R of AAV2 VP1) in their respective proteins. Among these residues, changes in 450S (GPV)/N(NGPV), 451R, and 581Q would affect the recognition of receptors, as they are situated in the vicinity of the receptor-binding sites [4]. Further analysis revealed that position 450 in NGPV VP1 mutated to N with a larger side chain than S in GPV VP1. This altered the spatial positions of 450N and 451R and the amino acids that interact with them: positions of 450N and 451R exchanged due to rotation, with 450N turned outward as the original space fitting 450S of GPV VP1 could not accommodate it and 451R turned inward which was on the protein surface initially. In GPV VP1, 450S and 451R interacted with 581Q and 582N, while in NGPV VP1, the interaction with 581Q and 582N was only 451R, owing to the mutation of 450N. Therefore, changes in the position and conformation of 450N are most likely the genetic basis of GPV recognized by duck-derived cells [32], which has not been reported in previous studies. Cross-species transmission caused by a single amino acid substitution has been confirmed in canine parvoviruses [33]. Therefore, parvoviruses with smaller genomes are prone to compress their functional domains to fewer amino acids, suggesting that more attention should be paid to other regular mutations conserved in different viruses or genotypes.

Considering the immunological cross-recognition between MDPV and GPV [34], GPV yolk Abs were initially used for emergency treatment of its distinct lineage, NGPV, which was gradually replaced by NGPV Abs, given their low effect. In this event, the mutation rate of viral genes, especially antigenic epitopes, may be faster to escape recognition by Abs. Therefore, NGPV exhibited an evolutionary rate comparable to that of RNA viruses [35], which was approximately 10 times faster than that of GPV and MDPV. The conservatism of epitopes was crucial to vaccine availability; therefore, we obtained the genetic evolution of NGPV prevalent in different periods and analyzed its impact on Ab recognition. There were 11 regular amino acid substitutes between NGPV and GPV, of which half (positions 553, 555, 573, 594, and 617) located in NS1 are components of B cell epitopes [36]. As the binding regions of the receptor and Ab present a certain overlap in the parvovirus [31], position 450, the only distinguishing site between NGPV and GPV on VP1, is most likely to affect Ab recognition. In the D271 and D278 infection experiments, GPV and NGPV were used as antigens to determine Ab titers, but precipitation lines were observed only when NGPV was used, indicating that amino acid substitutions affected the mutual recognition of Abs and antigens while partially explaining the unsatisfactory results of treating NGPV infection using GPV yolk Abs [37]. Focusing on different genotypes/subtypes of NGPV, compared with NGPV Ib prevalent during the early period, NGPV Ia and II exhibited two regular mutations (261 and 498) on VP1, in which position 498 was the C-terminal residue of the epitope recognized by monoclonal Ab against AAV2 [31]. Until recently, NGPV Ia circulating in China alone indicated that this subtype had a greater capacity to escape Ab recognition than NGPV II. Among the 11 regular mutations between NGPV Ia and II, positions 41, 142, 144, and 534, located in VP1, are components of different epitopes in GPV. These substitutions likely affect the efficacy of Abs. Although other mutations have not yet been confirmed, the amino acids commonly found in epidemic strains (NGPV Ia) should be selected for vaccine development. We believe vaccines or Abs developed based on NGPV Ia could achieve better prevention and control effects for a considerable period. However, in ducks challenged with D271 and D278, persistent infection was observed, and complete eradication of the virus was not achieved, even when targeting Abs at high levels. This result is consistent with the conclusion of Wang et al. [21]. Hence, to achieve better prevention and control, the coordinated application of humoral and cellular immunity may be a better strategy. With the slow progress of research on epitope identification, live attenuated vaccines are currently the most suitable type of vaccine for large-scale applications, which highlights the attenuation of the mechanism of virulence.

In the early period of NGPV circulation in China, the morbidity and mortality rates were 10–30% and 2–5%, respectively. Infected ducks presented typical symptoms, such as short beaks, protruding tongues, growth retardation, joint swelling, necrosis, and bleeding in multiple tissues and organs, including the heart and pancreas [9]. However, in 2022–2023, not only did the clinical symptoms change to just stunting and joint swelling manifested in ducks infected with NGPV in Shandong province, but the pathogenicity was also significantly attenuated, with the rates of morbidity and mortality reduced to 5% and 1%, respectively. In animal challenge experiments conducted with D271 and D278, no ducks died, and only two exhibited growth restriction during the normal observation period (approximately 30 days) [21]. Although bleeding points were observed in the intestines of the two ducks, no obvious lesions were observed on biopsy (pathological sections are not shown). The pathogenicity of NGPV prevalent in Shandong province was significantly attenuated, but the functional proteins or sites remain unclear. Therefore, we conducted a mutation analysis of NGPV genotypes circulating in the early and recent periods. Between NGPV Ia and Ib, there are three regular amino acid substitutions, among which position 159, the genetic marker for NGPV Ia, is located at the N-terminus of NS1, the determining region of viral pathogenicity. Meanwhile, positions 261 and 498 are located on the surface of VP1, with position 498 seated near positions 477 and 480, which are receptor-binding sites [38]. The VP and NS proteins can affect the virulence and pathogenicity of parvoviruses, but the known functional sites are concentrated in the receptor-binding region (AAV2, CPV, and FPV) and the natural recombination region (MDPV) in the VP protein [2,39,40] and two ends of the NS protein [41], respectively. Thus, the three sites, especially positions 159 and 498, were the most likely residues to cause differences in pathogenicity, although this remains to be confirmed. We also conducted a systematic mutation analysis of other prevalent strains at different periods; however, the lack of data on their pathogenicity made determining or predicting the role of regular mutations not possible. The reduction in virulence and pathogenicity is more conducive to the transmission and spread of viruses [27,42], and NGPV Ia, with attenuated pathogenicity, is currently the dominant genotype prevalent in China. Therefore, NGPV Ia would possibly remain the dominant genotype in Shandong and over a considerable period in the future. The development of attenuated vaccines aimed at NGPV Ia should be expedited, and the noted locations above should be examined as potential targets.

The above analysis indicates mutations preferred being located in VP1 when NGPV genes of different genotypes or different period were compared, which differed from the comparison between NGPV and GPV. There were seven regular amino acid substitutions located in VP1 in comparison between NGPV I and II, which were completely conserved in each genotype. Two regular differential sites (positions 261 and 498) between NGPV isolates prevalent in the early period and after 2017 were located in VP1; most of the 11 regular mutation sites between the two genotypes (subtypes) divergent after 2017 were also positioned on the VP1 protein. These results are consistent with the characteristics of parvoviruses [43]. In contrast, internal proteins would remain stable unless viral fitness, a basic condition for viral function, is threatened [44]. Meanwhile, more T cell epitopes are common in internal than external proteins [45,46]; thus the NGPV NS1 protein, also possessing B cell epitopes, may be more suitable for the development of next-generation vaccines than the VP1 protein.

## 5. Conclusions

Collectively, prevalence of NGPV in China has undergone three stages, with different dominant genotypes circulating in each stage. The five NGPV strains isolated in this study belonged to NGPV Ia, the dominant subtype overall. The VP1 protein is the leading factor in viral evolution, whereas the NS1 protein has a different evolutionary pattern. Compared with GPV, NGPV exhibits a more rapid evolutionary rate and produces regular mutations at different times and functional levels. Their location and the resultant changes in conformation were most likely the genetic basis for cross-species transmission, immune escape, and pathogenic alterations of the virus, which preliminarily confirmed pathogenicity. Given the persistent infection and high variability of surface proteins, attenuated vaccines targeting the prevalent genotype may be suitable for addressing current NGPV epidemic, whereas third-generation vaccines targeting NS1, with the coordinated application of humoral and cellular immunity, are the most efficient means of preventing and controlling NGPV.

## Figures and Tables

**Table 1:** Primers used for amplification of isolated novel goose parvovirus.

No.	Sequence (5′–3′)	Position	Product Size (bp)
1	1F ^1)^: CTYATTGGAGGGTTCGTT	1–191	191
	1R ^2)^: GCATGCGCGTGGTCAACCTAACA		
2	2F: GCATGCCGCGCGGTCAGCCCAAT	186–1575	1390
	2R: AGGGTACAGCATGGRCAATAG		
3	3F: CACCACCGGGAAGACCAACAT	1526–2487	962
	3R: CAGCTTTCAGATTCCGCCACG		
4	4F: GASTGGTATGAGACTGCAGCCG	2442–3112	671
	4R: TYTCCCATCCATTGGGAATCG		
5	5F: GGGGGTGCCGATGGAGTGGGTA	3048–4228	1181
	5R: TTCTGCCACACCATKCCWGGTA		
6	6F: KWTAGCTAAAGATCCATACAGA	4022–5052	1031
	6R: CTTATTGGAGGGTTCGTTCGTTC		

^1)^ F, forward primer; ^2)^ R, reverse primer.

**Table 2:** Biological characteristics of the five newly isolated novel goose parvovirus.

Name	Collection Time	Collection Location	Duck Variety	Age (d)	Death Time of Embryo	ELD_50_/ 0.2 mL	Accession No.
D187	13 November 2022	Jinan	Cherry Valley	10	96 h (3th generation)	10^−2.318^	PQ807633
D271	11 March 2023	Heze	Cherry Valley	18	48 h (3th generation)	10^−6.32^	PQ807634
D272	11 March 2023	Yantai	Cherry Valley	36	96 h (4th generation)	10^−3.50^	PQ807635
D274	18 March 2023	Linyi	Cherry Valley	10	72 h (4th generation)	10^−4.42^	PQ807636
D278	18 March 2023	Binzhou	Cherry Valley	14	144 h (3th generation)	10^−6.16^	PQ807637

**Table 3:** Positive selection sites and the change in characteristic in novel goose parvovirus.

Protein	Residue	MEME ^1)^ *p* Value	SLAC ^2)^ *p* Value	FUBAR ^3)^ *Positively Probability*	Major	Characteristic	Mutation	Characteristic
NS1	293	0.07		0.752	L	non-polar	Q	polar
	498	0	0.119	0.992	R	polar	E/K/T	pola
	602		0.198	0.977	A	non-polar	T	polar
VP1	93	0.07		0.702	A	non-polar	S	polar
	180	0.06		0.769	V	non-polar	A	non-polar
	261	0.03		0.854	S	polar	A	non-polar
	453	0.02		0.714	A	non-polar	T/V	polar/non-polar
	468	0		0.733	R	polar	C/H	polar

^1)^ MEME: mixed-effects model of evolution; ^2)^ SLAC: single-likelihood ancestor counting; ^3)^ FUBAR: fast unconstrained Bayesian approximation; Significant difference: MEME *p* < 0.1, SLAC *p* < 0.2, FUBAR *positively probability* ≥ 0.7.

**Table 4:** Nucleotide and amino acid sequence homology across five newly and four previously identified novel goose parvovirus and goose parvovirus (%).

Strain	SDLC19 (Ia)					QH15 (Ib)					AHAU30 (II)					Virulent B (GPV)				
	Nucleotide			Amino Acid		Nucleotide			Amino Acid		Nucleotide			Amino Acid		Nucleotide			Amino Acid	
	G ^1)^	NS1	VP1	NS1	VP1	G	NS1	VP1	NS1	VP1	G	NS1	VP1	NS1	VP1	G	NS1	VP1	NS1	VP1
D187	99.5	99.6	99.8	99.4	99.6	97.9	98.9	98.7	98.4	97.8	97.7	98.9	97.3	98.4	97.0	94.8	96.7	96.1	96.8	97.7
D271	99.4	99.6	99.8	99.0	99.6	97.8	98.9	98.7	98.1	97.8	97.6	98.9	97.4	98.1	97.0	94.8	96.6	96.1	96.5	97.7
D272	99.5	99.8	99.7	99.5	99.3	97.9	99.1	98.6	98.6	97.5	97.7	99.1	97.2	98.6	96.7	94.8	96.8	96.0	97.0	97.4
D274	99.0	99.8	99.9	99.5	99.7	97.5	99.1	98.8	98.6	98.0	97.2	99.1	97.4	98.6	97.1	94.5	96.9	96.2	97.3	97.8
D278	99.2	99.6	99.1	98.9	98.4	97.6	98.9	98.1	97.9	96.9	97.3	98.9	96.6	97.9	95.8	94.6	96.7	95.5	96.7	96.5

^1)^ G, genome.

**Table 5:** Characteristic mutations present in two proteins of novel goose parvovirus.

Virus/Genotype/Subtype	NS1										VP1									
	50	131	140	159	468	553	555	573	594	617	41	89	142	144	207	210	261	450	498	521
NPGV ^1)^	T	R	S		I	K	T	Q/K	Y	A								N/D		
GPV ^2)^	I	K	A		V	R	N	E	D	V								S		
NPGV I											D	L	E	I	M	S				H
NGPV II											A	Q	D	V	L	A				Y
NPGV Ia				V													A		S	
NGPV Ib				L													S		N	
NPGV Ia				V																
NPGV Ib/II and GPV				L																

^1)^ NGPV: novel goose parvovirus; ^2)^ GPV: goose parvovirus.

**Table 6:** Characteristic mutations of novel goose parvovirus in different periods and genotypes.

Items	NS1	VP1											
	159	41	89	116	142	144	207	210	261	498	520	521	534
NGPV ^1)^ after 2017 (NPGV Ia/II)									A	S			
NGPV during 2015–2017 (NPGV Ib)									S	N			
NPGV Ia	V	D	L	Y/H	E	I	M	S			A	H	S
NPGV II	L	A	Q	Q	D	V	L	A			T	Y	I

^1)^ NGPV: novel goose parvovirus.

## Data Availability

All the sequenced strains used in this study are available in the National Center for Biotechnology Information (NCBI).

## Abbreviations

The following abbreviations are used in this manuscript:

NGPV	Novel goose parvovirus
SBDS	Short beak and dwarfism syndrome
GPV	Goose parvovirus
MDPV	Muscovy duck parvovirus
ORF	Open reading frames
UTR	Untranslated regions
ITR	Inverted terminal repeats
MLTF	Major late transcription factor
RBS	Rep protein binding site
ATF/CREB	Activating transcription factor/cAMP response element binding protein
bp	Base pair
aa	Amino acid
SF3	Helicase superfamily 3
PLA2	Phospholipase A2
NLS	Nuclear localization signal
Ab	Antibody
AGP	Agar gel precipitation
